# LH prevents AMH action via receptor downregulation in human primary granulosa lutein cells

**DOI:** 10.1007/s10815-025-03635-x

**Published:** 2025-08-28

**Authors:** Francesca Liuzzi, Clara Lazzaretti, Serena De Carlini, Alice Michelini, Manuela Simoni, Livio Casarini, Antonio La Marca

**Affiliations:** 1 Department of Medical and Surgical Science for Children & Adults, University of Modena and Reggio Emilia, Policlinico di Modena, Modena, Italy; 2 Unit of Endocrinology, Department of Biomedical, Metabolic and Neural Sciences, Baggiovara Hospital, University of Modena and Reggio Emilia, Modena, Italy; 3 Center for Genomic Research, University of Modena and Reggio Emilia, Modena, Italy; 4 Department of Medical Specialties, Azienda Ospedaliero-Universitaria di Modena, Baggiovara Hospital, Modena, Italy

**Keywords:** Anti-Müllerian hormone (AMH), Luteinizing hormone (LH), Granulosa lutein cells, AMHR2, Folliculogenesis, SMAD signaling

## Abstract

**Purpose:**

Gonadotropins and anti-Müllerian hormone (AMH) regulate reproductive development and ovarian function. While AMH plays a well-established role in early folliculogenesis by counteracting gonadotropins, luteinizing hormone (LH) becomes more active later, during the antral phase, when granulosa and theca cells express their respective receptors. There are hints suggesting the existence of an interplay between these hormones regulating granulosa cell functions at late stages of the folliculogenesis., but the mechanisms remain unclear. In this context, we explored whether gonadotropin LH can modulate AMH action in cells of ovarian origin.

**Methods:**

Primary human granulosa lutein cells isolated from in vitro fertilization (IVF) patients were pretreated with recombinant LH, followed by stimulation with recombinant AMH. AMH receptor type II (AMHR2) expression, AMH signaling activation, and the expression of AMH-responsive genes were assessed through RT-PCR, ELISA, and Western blotting. The involvement of the LH receptor was confirmed using siRNA-mediated knockdown.

**Results:**

Our findings showed that LH treatment downregulates AMHR2 transcripts and protein, impairing AMH-induced phosphorylation of small mothers against decapentaplegic (SMAD) 1, 5, and preventing osterix (*OSX*) and matrix metalloprotease 2 (*MMP2*) gene expression.

**Conclusions:**

These data are consistent with the possible interplay between gonadotropins and AMH in cells from antral/luteal stages and provide the molecular basis for further studies evaluating the impact of LH in modulating AMH signaling and follicular responsiveness during the antral stage.

## Introduction

Anti-Müllerian hormone (AMH) is a member of the transforming growth factor beta (TGF-β) superfamily. It acts through its “type II membrane receptor” (AMHR2), triggering the phosphorylation of small mothers against decapentaplegic (SMAD) 1, 5, or 8 proteins [1, 2]. These molecules activate SMAD 4, which translocates to the nucleus and leads to target gene transcription [2]. SMAD-dependent genes are osterix (*OSX*), matrix metallopeptidase 2 (*MMP2*), and WNT inhibitory factor (*WIF1*), and were mainly described in the context of the Müllerian duct regression [3].

In females, AMH may be produced by ovarian granulosa cells, where AMH secretion achieves maximal levels during pre-antral and early antral stages, while it decreases in the antral stage, during the gonadotropin-dependent stage of the follicular growth [4]. In fact, several studies demonstrated that AMH mediates autocrine and paracrine effects during the early stages of folliculogenesis [5], when it is produced by the growing follicles and negatively regulates the initial follicular recruitment [6]. Moreover, AMH acts in granulosa cells of developing follicles in an autocrine manner, reducing the sensitivity to gonadotropins and attenuating aromatase synthesis [7, 8].

Clinical data suggested the existence of a link between AMH, gonadotropin levels and ovarian physiology in the antral stage [9, 10], and AMH was proposed as a reliable diagnostic indicator for ovarian follicular reserve surrogate to the antral follicle count [11–13]. In fact, AMH serum levels are relatively low in patients with premature ovarian failure, who are characterized by low gonadotropin levels. On the contrary, AMH and luteinizing hormone (LH) levels are high in patients affected by polycystic ovary syndrome (PCOS) [14]. These studies indicate that LH might play a significant role in promoting early ovarian follicular recruitment, growth and maturation, impacting AMH physiology. The hypothesis is clinically corroborated by the benefit of LH treatment in women affected by hypothalamic amenorrhea, who underwent improved antral follicular count, increased AMH levels and mature oocytes recruited [15]. All together, these studies provide the rationale for investigating the relationship between LH and AMH in ovarian cells, as an issue still poorly known [16].

The aim of this study in vitro is to investigate whether LH modulates AMH action in ovarian granulosa cells.

## Materials and methods

### Experimental design

Granulosa cells collected from in vitro fertilization (IVF) patients were cultured and treated with recombinant LH before evaluating AMH production and AMHR2 expression. The functional effect of LH-induced modulation of AMHR2 levels was assessed under cell treatment with recombinant AMH. In these cells, AMH-induced SMAD phosphorylation and target gene expression were evaluated.

### Isolation and culture of human granulosa cells

Human granulosa lutein cells were isolated from follicular fluids collected from women undergoing IVF at the Department of Obstetrics and Gynecology of Modena University Hospital, with ethical approval and informed written consent (nr. 2210 19^th^ June 2014, Modena, Italy). Follicular fluids from 2–3 patients were pooled and centrifuged (800 ×g, 30 min) by discontinuous Percoll gradient (GE Healthcare, Little Chalfont, UK). The granulosa cell-enriched layer was collected

[17] and resuspended in Dulbecco’s Modified Eagle’s (DMEM)/Ham’s F-12 medium containing 10% fetal bovine serum, 2 mM glutamine, 100 IU/ml penicillin, 100 µg/ml streptomycin, and 0.25 µg/ml amphotericin B (all reagents were purchased from Gibco, Life Technologies Co., Carlsbad, CA, USA). Cells were seeded in a 24-well plate at a concentration of 1×10^5^ cells per well, cultured 6 days to recover the optimal metabolic state required for in vitro experiments and serum-starved overnight before treatments. Experiments were performed using recombinant LH (Luveris; Merck KGaA, Darmstadt, Germany) and recombinant AMH (R&D Systems, Minneapolis, MN, USA).

### siRNA trasfection protocol and treatments

Granulosa cells were exposed 48 h to 10 nM siRNA against luteinizing hormone/choriogonadotropin receptor (*LHCGR*; s8163, Ambion - Thermo Fisher Scientific, Waltham, MA, USA) or to 10 nM of a control siRNA, using TransIT-LT1 as transfection reagent (Mirus Bio, Madison, WI, USA) and according to the manufacturer’s instructions. SiRNA-treated cells were exposed to increasing LH concentrations (100 pM, 1 nM, 10 nM, corresponding to the 6600–2.2 mIU/ml dose range) or DMEM/F-12 (negative control) 24 or 48 h.

### Gene expression analysis

Total RNA was extracted using TRI-Reagent (Sigma-Aldrich, Merck KGaA), according to the manufacturer’s recommendations [18]. RNA concentration and quality were determined by Nanodrop ND-1000 (Thermo Fisher Scientific), then reverse-transcribed using the High-Capacity RNA-to-cDNA™ Kit (Thermo Fisher Scientific). Gene expression analysis was performed by real-time PCR using the Applied Biosystem Real-Time PCR System (Thermo Fisher Scientific), while reactions were prepared using PowerTrack™ SYBR Green Master Mix following the manufacturer’s recommendations (Thermo Fisher Scientific). *AMH* and *AMHR2* gene expression levels were determined using a protocol previously described [19], and results were normalized against the ribosomal protein S7 (*RPS7*) gene expression [20] using the 2^−ΔΔCt^ method [21]. Primers used were the following: *AMH* forward primer (Fwd) 5′-TACACCTGGAGGAAGTGACC-3′; *AMH* reverse primer (Rev) 5′-AGGGTACAGCACCAGCAG-3′; *AMHR2* Fwd 5′-GAGGTCATGC AGTGGTTTGG-3′; *AMHR2* Rev 5′-GGCTGGCAGTGATAAATCGG-3′; OSX Fwd 5′-GCCAGAAGCTGTGAAACCTC-3′; OSX Rev 5′-GCTGCAAGCTCTCCATAACC-3′; MMP2 Fwd 5′-GAGCTCTATGGGGCCTCTCC-3′; MMP2 Rev 5′-CGTCACAGTCCGCCAAATGA-3′;

*RPS7* Fwd 5′-AATCTTTGTTCCCGTTCCTCA-3′; *RPS7* Rev 5′-CGAGTTGGCTTAGGCAGAA-3′.

### Evaluation of AMH levels

Cells were exposed 24 h to treatments and AMH levels secreted in culture medium were measured by enzyme-linked immunosorbent assay (ELISA; ab267629; Abcam, Cambridge, UK) according to the manufacturer’s instructions. Optical density (OD) values were measured at 450 nm and protein concentration was extrapolated from the standard curve. The hormone concentrations were normalized considering the total protein content determined by Bradford assay.

### Western blotting analysis

AMHR2 protein expression, and phosphorylation levels of SMAD 1/5, were evaluated by Western blotting. In siRNA-treated and untreated cells, AMHR2 expression was assessed after 48-h exposure of samples to 0.1, 1.0, and 10 nM LH. Phospho-SMAD (pSMAD) was evaluated in cells exposed 48 h to 10 nM LH, and treated 15 and 30 min by 100 ng/ml AMH. Treated cells were treated by lysis buffer (50 mM Tris-EDTA, 150 mM NaCl, 0.1% SDS, 0.5% sodium deoxycholate, and 0.1% Triton X-100) containing protease and phosphatase inhibitors (Halt™ Protease and Phosphatase Inhibitor Cocktail; Thermo Fisher Scientific). Protein concentration of cell lysates was measured by bicinchoninic acid (BCA) Protein Assay Kit (Thermo Fisher Scientific), according to the manufacturer’s protocol. Protein samples were supplemented by 4X Bolt™ LDS Sample Buffer and 10X Bolt™ Sample Reducing Agent (Thermo Fisher Scientific) before being boiled at 100° for 5 min for protein denaturation. Equal amounts of proteins (20 μg) for each sample were run on 4–12% SDS-polyacrylamide gel (SDS-PAGE) at 140 V and transferred onto a nitrocellulose membrane (Thermo Fisher Scientific) at 100 V, 75 min. For the analysis of AMHR2 expression, membranes were incubated overnight at 4 °C in a 5% non-fat dry milk-blocking solution prepared in Tris-buffered saline, 0.05% Tween® 20 Detergent (TBST), while membranes for the analysis of SMAD phosphorylation were incubated 2 h at room temperature with 2% of bovine serum albumin blocking solution prepared in the same buffer. Membranes were subsequently incubated overnight at 4 °C with the following primary antibodies: mouse anti-AMHR2 antibody (1:800 NBP1-05491, Novus Biologicals, Centennial, CO, USA), rabbit anti-pSMAD 1/5 (1:500 BK9516S, Cell Signaling Technology, Danvers, MA, USA), mouse anti-β-ACTIN (1:5000 A3854, Sigma-Aldrich, Merck KGaA), and rabbit anti-VINCULIN (1:1000 #700062, Thermo Fisher Scientific). Membranes were then incubated 2 h at room temperature with proper secondary horseradish peroxidase-conjugated antibodies (1:10000 of G21040 goat anti-mouse and G21234 goat anti-rabbit, respectively, Thermo Fisher Scientific). An enhanced chemiluminescent (ECL) solution (Amersham Corp, Arlington Heights, IL, USA) was used for signal detection. The images were acquired by Chemi DocTM XRS 2015 (Bio-Rad Laboratories Inc., Hercules, CA, USA) image analysis system. Densitometric analysis was performed using Image Lab software and normalized using β-ACTIN or VINCULIN signals.

### Statistical analysis

All data are presented as means ± standard deviation (SD). Statistical analyses were performed using GraphPad Prism 9 (Dotmatics, Boston, MA, USA). D'Agostino and Pearson test was used to evaluate the normal distribution of data. Results were analysed by Kruskal-Wallis test and Dunn’s post hoc test. Differences were considered as significant with *p* < 0.05.

## Results

### LH does not impact AMH gene expression and protein secretion

We evaluated the effect of LH treatment on *AMH* gene and protein expression. To this purpose, granulosa cells were treated 24 h with 0.1–10 nM LH before *AMH* gene expression analysis by real-time PCR, and AMH protein analysis by ELISA.

**Fig. 1:**
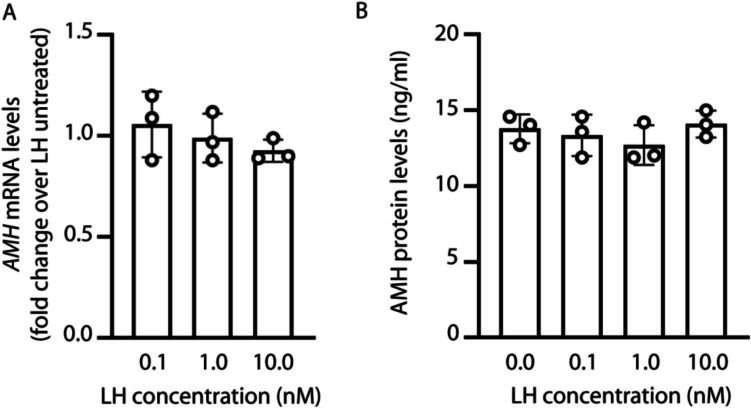
Analysis of AMH mRNA and protein levels. Granulosa cells were treated with LH before measuring AMH transcripts and hormone levels by real-time PCR and ELISA, respectively. **A** *AMH* mRNA analysis. Results were calculated with the 2^-ΔΔCt^ method and normalized over those from LH untreated samples, which have values equal to 1 (not showed). **B** Analysis of AMH protein levels. Statistical analysis was performed using the Kruskal-Wallis test; *p* ≥ 0.05

No significant differences in *AMH* gene (Fig. 1A) and protein expression levels (Fig. 1B) between LH-treated *vs* untreated cells were found (Kruskal-Wallis test; *p* ≥ 0.05), indicating that the gonadotropin does not modulate the hormone production and secretion by granulosa cells.

### LH treatment downregulates AMHR2 levels

To evaluate if LH could modulate AMHR2, granulosa cells were treated 48 h with 0.1–10 nM of the gonadotropin before measurement of receptor mRNA and protein levels. Expression values were normalized over those of LH-untreated samples.

**Fig. 2:**
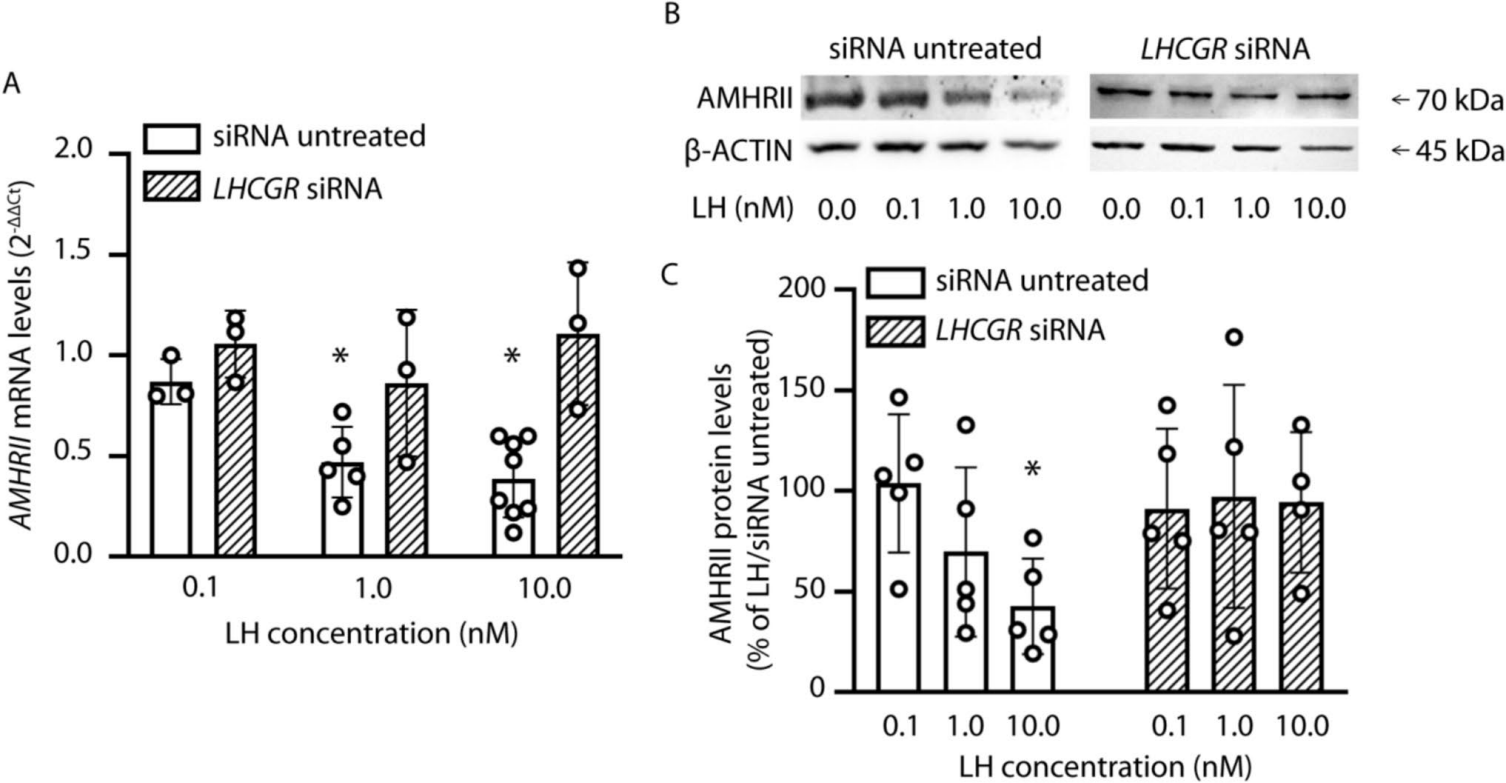
LH-induced inhibition of AMHR2 expression. Granulosa cells were exposed 48 h to LH, in the presence or in the absence of siRNA against LHCGR, and AMHR2 mRNA and protein levels were evaluated by real-time PCR and Western blotting, respectively. **A** *AMHR2* mRNA analysis. Results were calculated using the 2^-ΔΔCt^ method considering the *RPS7* gene expression and normalized over those from LH/siRNA untreated samples, which have values equal to 1 (not showed). * = significantly different to LH untreated samples; Kruskal-Wallis test, *p* < 0.05. **B** Western blotting picture of AMHR2. β-ACTIN served as a normalizer. **C** Densitometric quantification of AMHR2 Western blotting bands. Results were normalized as the percentage of signals from LH/siRNA untreated samples, which have values equal to 100% (not showed). * = significantly different *versus* LH untreated samples; Kruskal-Wallis test, *p* < 0.05

Real-time PCR analysis of *AMHR2* mRNA levels revealed dose-dependent reduction of receptor transcripts compared to those from untreated cells (Fig. 2A; Kruskal-Wallis test; *p* < 0.05). The dependence on LH of *AMHR2* transcript decrease was demonstrated in siRNA-treated cells, where the gonadotropin failed to induce receptor mRNA downregulation under LHCGR depletion (Fig. 2A; Kruskal-Wallis test; *p* ≥ 0.05).

AMHR2 protein analysis was performed by Western blotting (Fig. 2B), and the densitometric semi-quantification confirmed that 10 nM LH induces reduction of receptor expression (Fig. 2C; Kruskal-Wallis test; *p* < 0.05). LHCGR depletion by siRNA prevented AMHR2 downregulation by LH (*p* ≥ 0.05).

### LH inhibits AMH-mediated SMAD 1/5 phosphorylation

**Fig. 3:**
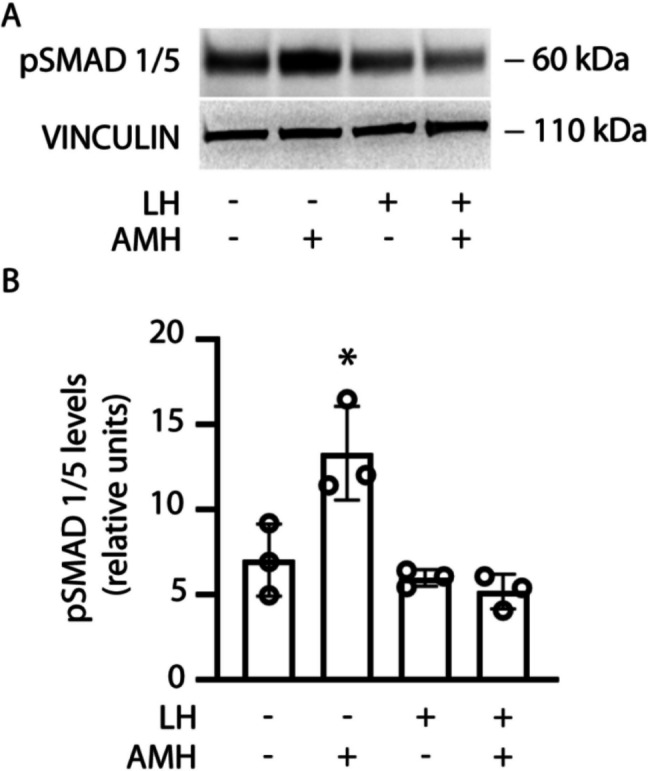
Reduction of AMH action by LH-induced AMHR2 downregulation. 48-h LH-treated granulosa cells were exposed to AMH and SMAD 1/5 phosphorylation was evaluated by Western blotting. **A** Western blotting picture of AMH-induced pSMAD activation. VINCULIN was the loading control. **B** Densitometric analysis of pSMAD Western blotting bands. * = significantly different *versus* AMH untreated samples unexposed to LH; Kruskal-Wallis test, *p* < 0.05

Since LH downregulates AMHR2 expression levels (Fig. 2), we investigated the effects of AMH under gonadotropin-mediated receptor downregulation. To this purpose, granulosa cells were treated 48 h with 10 nM LH before to be exposed 15 min to recombinant AMH. The phosphorylation of SMAD 1/5 was evaluated by Western blotting (Fig. 3).

In cell treated with LH, Western botting analysis revealed that AMH induced pSMAD activation, while it was not in cells treated with the gonadotropin (Fig. 3A). The densitometric analysis confirmed that cell treatment with LH is linked to the failure of AMH-induced pSMAD activation (Fig. 3B).

### LH prevents AMH-mediated gene expression

**Fig. 4:**
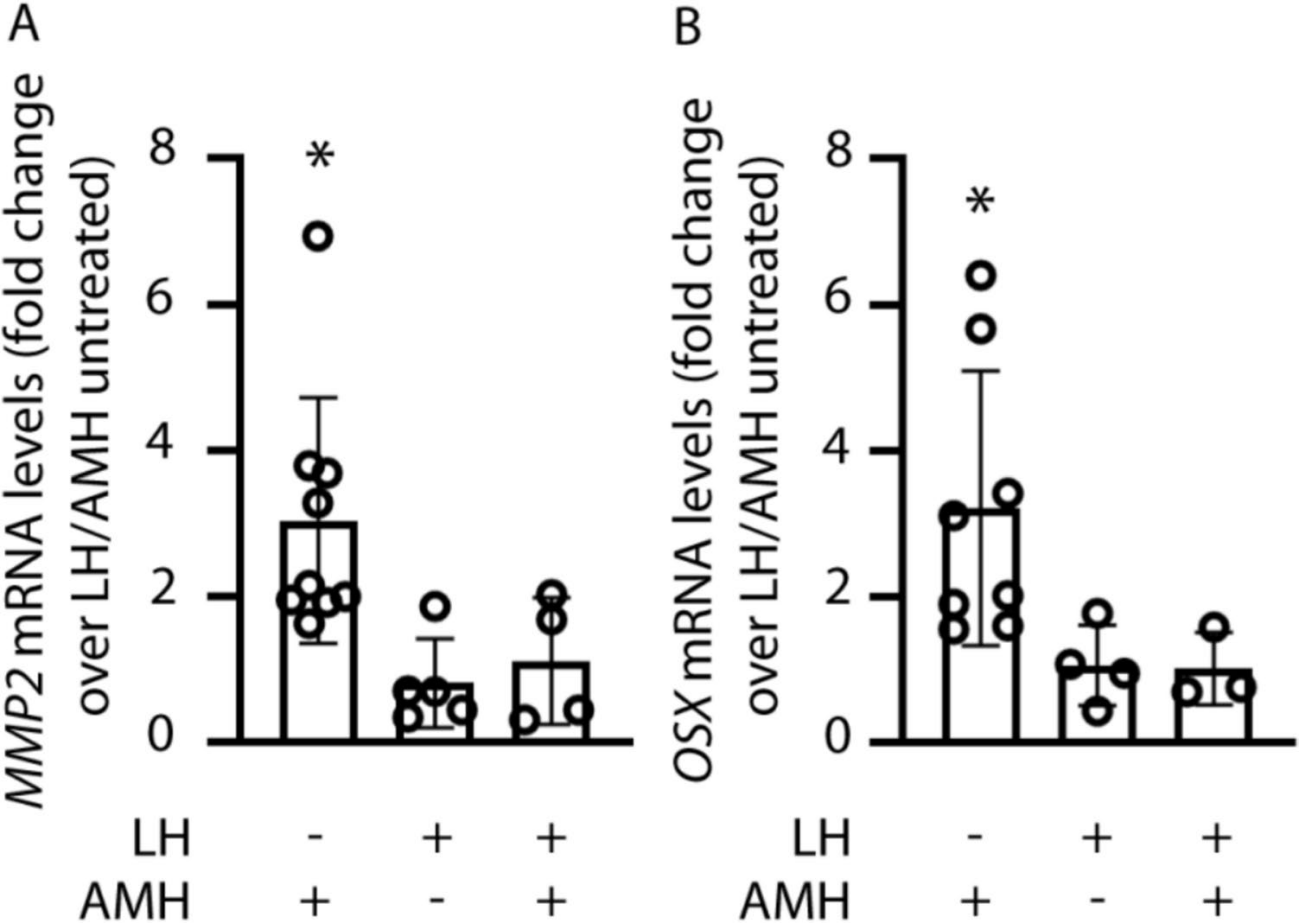
LH-dependent prevention of AMH-induced gene expression. Granulosa cells were treated 48 h with 10 nM LH, and *MMP2* and *OSX* gene expression levels were evaluated by real-time PCR after further 24-h exposure to 100 ng/ml AMH. Results were calculated using the 2^-ΔΔCt^ method, using the *RPS7* as the housekeeping gene, and indicated as fold-increase against LH/AMH untreated samples, which have values equal to 1 (not showed). A) *MMP2* gene expression analysis. B) Analysis of *OSX* gene expression levels. * = significantly different *versus* LH/AMH untreated samples; Kruskal-Wallis test, *p* < 0.05

We evaluated if the LH-dependent inhibition of AMRHII expression levels (Fig. 2) and signalling (Fig. 3) impacts AMH target gene expression. Therefore, granulosa cells were exposed 48 h to LH and the AMH-induced expression of *OSX* and *MMP2* genes was evaluated (Fig. 4).

Real-time PCR analysis revealed that 24 h of exposure to 100 ng/ml AMH significantly increased the expression of both *MMP2* (Fig. 4A) and *OSX* (Fig. 4B) in LH-untreated cells (Kruskal-Wallis test; *p* < 0.05), whereas it was not in cells exposed to the gonadotropin before AMH treatment (*p* ≥ 0.05).

## Discussion

We demonstrated that granulosa cell exposure to LH negatively modulates AMH action. The gonadotropin decreased AMHR2 transcript and protein levels, and this event is linked to impaired action of AMH at the intracellular level, preventing SMAD phosphorylation and hormone target gene expression. These data point out the relevance to elucidate the connection between gonadotropins and AMH in the antral stage, since these molecules are indicative of the ovarian reserve and response to clinical treatments [22].

Our results corroborate studies supporting that the actions of AMH and gonadotropins are linked and may collaborate in the modulation of the antral follicle physiology in women [23]. AMH may regulate the hypothalamus-pituitary-gonadal axis via direct action on gonadotropin-releasing hormone (GnRH) neurons, thus impacting gonadotropin secretion. However, since ovarian AMH secretion was demonstrated [24], the knowledge that AMH had only male-specific functions has been extensively revised over the last three decades, and studies focused on the action of this hormone in the female gonad [23]. While the direct action of AMH in ovarian cells at the pre-antral stages is well known, where the hormone prevents follicle-stimulating hormone (FSH) action [25], less is known about its possible role during the antral stage. A study in isolated follicles from primates revealed that AMH could have a dual role [26], since it directly supports pre-antral follicle growth, while acts as a negative regulator of follicle growth in the antral stage [27]. These data were corroborated by a study in human primary granulosa lutein cells, that suggested AMH could inhibit FSH-induced steroidogenesis via downregulation of genes coding key enzymes [28]. All together, these studies convergently suggest that AMH exerts opposed action to FSH in the antral stage, while data elucidating the network between LH and AMH are poor.

In the antral stage, LH supports androgen synthesis, dominant follicle maturation and ovulation, and it is responsible for later luteinization and progesterone synthesis [29]. Moreover, it has been recently hypothesized that the hormone might play a role in the late pre-antral/early antral stage when it would support the recruitment of the antral pool [15] via an unknown molecular mechanism. Although this hypothesis requires further studies to be deepened, it provides a new point of view on female gonadal physiology, since it is assumed that the antral follicle pool recruitment is supported by FSH [30, 31]. Moreover, it could lead to new approaches to infertility treatments, whose outcome could benefit from LH administration [32]. Our results could find a place in this context, since we found that AMH action is inhibited by LH treatment, potentially relieving the opposed action to FSH in the antral stage. Therefore, we may speculate that antral follicle maturation may benefit of a dual LH action [20]; the first would be due to its direct upregulation of intracellular survival signals activated in granulosa cells [19], while the second would consist in the indirect effect exerted via AMHR2 downregulation demonstrated herein.

This study has also limitations and further investigations are required before achieving conclusions. We have used primary granulosa cells, which are of human ovarian origin and express some markers of human ovarian antral/lutein cells, i.e. gonadotropin and AMH receptors and steroidogenic enzymes [19]. However, they could not fully reflect the metabolic state of antral granulosa cells. Moreover, the possible impact of other molecules potentially modulating the effects of LH and absents in the in vitro environment should be considered as well.

## Conclusions

In conclusion, our results support the existence of a regulatory network connecting the effects of AMH and LH in ovarian cells. At the net of experimental limitations, these data reflect previous observations by which LH could exert beneficial effects on follicular maturation.

## Data Availability

All data are available along with this manuscript. Further requests may be addressed to the corresponding author.

## Declarations

### Attestation Statement

The data presented in this manuscript are original, have not been published elsewhere, and are not under consideration for publication by any other journal. The original/raw data supporting the findings of this study are available and will be provided to the journal upon request.
